# Artificial intelligence and radiographer preliminary image evaluation: What might the future hold for radiographers providing x‐ray interpretation in the acute setting?

**DOI:** 10.1002/jmrs.821

**Published:** 2024-09-20

**Authors:** Clare Rainey

**Affiliations:** ^1^ School of Health Sciences Ulster University Belfast UK

## Abstract

In a stretched healthcare system, radiographer preliminary image evaluation in the acute setting can be a means to optimise patient care by reducing error and increasing efficiencies in the patient journey. Radiographers have shown impressive accuracies in the provision of these initial evaluations, however, barriers such as a lack of confidence and increased workloads have been cited as a reason for radiographer reticence in engagement with this practice. With advances in Artificial Intelligence (AI) technology for assistance in clinical decision‐making, and indication that this may increase confidence in diagnostic decision‐making with reporting radiographers, the author of this editorial wonders what the impact of this technology might be on clinical decision‐making by radiographers in the provision of Preliminary Image Evaluation (PIE).

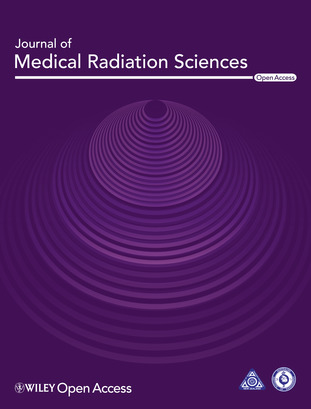

## Introduction

Health care services around the world are becoming increasingly stretched. This may be due to many factors, such as increasing population age, increasing burden of sickness and, importantly, limited human resources.^1^, ^2^, ^3^ Indeed, the most recent Royal College of Radiologists (RCR) census in the United Kingdom (UK) noted that there is a 30% shortfall of radiologists, predicted to increase to 40% by 2028 if no action is taken.^4^ Additionally, the increase in the rate of technological development in radiology, suggests that imaging is being increasingly utilised.^5^ This increase in data volume adds additional pressure on an already stretched workforce, and production of suitably qualified radiology staff does not hold pace with demand, with delays in the provision of radiology reports.^6^ Radiographers are ideally placed to help in both the formal and informal review and interpretation of radiographic images. This has been supported in the Getting it Right First Time (GIRFT) radiology report^7^ in the UK, where it is noted that, in some healthcare trusts in England, radiographers are providing final, formal ‘reports’ on 50% of plain projection radiographic images. However, the Report also recognises that significant staffing shortages in the radiography workforce mean that this may not be possible and that the practice of formal radiographer reporting in the UK varies widely. There are, however, means of using the expertise of the radiographer other than provision of an ‘official report’ which can reduce patient waiting times, improve accuracy and safeguard the patient. Furthermore, new advances in computer vision and Artificial Intelligence (AI) have led to developments in the use of these technologies for assistance in image interpretation and diagnostic decision‐making. These, also, have been proposed to reduce reporting turnaround times and increasing diagnostic accuracy.^7^, ^8^

## The Unique Advantages of Radiographer Image Evaluation

The clinical radiographer has the advantage of both production of the image, based on the clinical information provided by the referrer, and having the patient with them in the x‐ray room to gain further understanding of the clinical presentation, should the need arise. They are, therefore, ideally placed to provide an initial assessment of any pathology present on the images produced. In the UK and elsewhere, some means of radiographer identification of abnormality/pathology on radiographs (‘red dotting’) has been in place since the 1980s;^9^ however, problems with the lack of required information to explain what the radiographer was referring to meant that this system had limited usefulness.

In 2013, The Society and College of Radiographers in the UK published guidance for the provision of Preliminary Clinical Evaluation (PCE) or Preliminary Image Evaluation (PIE) as it is known in many other parts of the world.^10^ PIE describes a brief statement which provides additional information, in written format, to support the ‘red dot’. Harcus and Stephens (2021)^11^ report that emergency department clinicians and radiology reporters (reporting radiographers and radiologists) find a brief ‘bullet‐point’ comment of ‘What, Where and How’ in relation to the pathology is desirable. Despite the simplicity of the information desired, radiographers remain reticent to adopt PIE in their clinical practice with myriad reasons cited for this, such as accountability, lack of confidence, feelings of being ill‐prepared from an education and training perspective^12^ and time pressures.^13^ Specifically, a large Australian study by Brown et al.,^13^ published in this edition of the Journal of Medical Radiation Sciences (JMRS), investigates the impact of ‘workload’ on the accuracy of PIE and reports that whilst radiographers perceive that a lack of time impacts the accuracy of their PIE, this was in fact not the case in their setting.

## Why Is Initial Image Evaluation Important?

Alexander‐Bates et al.,^14^ noted that although a formal, definitive report remains the optimal standard, this may not always be provided in a timely manner for the efficient movement of the patient through the required pathway, especially in the emergency setting. Emergency departments are feeling the impact of staffing shortages also and the provision of an initial interpretation can increase diagnostic accuracy and help the various clinicians in the emergency department. A relatively recent study by Bachmann et al.,^15^ found that reporting radiographers (with 1 to 4 years' experience) in a clinical centre in Denmark were significantly less likely to make errors which would be detrimental to the patient compared to radiology and orthopaedic trainees, each working for some part of their routine clinical work in the emergency department. Whilst this study focussed on reporting radiographers, it indicates that with suitable training for frontline radiographers, improvements can be made in error reduction for patients. This training is usually part of pre‐registration training for radiographers in many countries. For instance, there is a requirement in the Standard of Proficiency for diagnostic radiographers for both the Health and Care Professionals Council (HCPC) in the UK and the Medical Radiation Practice Board of Australia (MRPBA) which states that practicing radiographers should be able to identify *and communicate* any urgent or unexpected findings to the referrer.^16^, ^17^

## Radiographer PIE Capabilities

Data gathered in the UK suggests that, despite impressive PIE performances of up to 92% accuracy, there may be a higher instance of false negative interpretations (sensitivity 80%, specificity 97%).^18^ This, of course, requires careful scrutiny due to the impact this may have on the patient. However, the incidence and implications of a false negative report are less investigated, despite this also having impact on the patient in terms of ‘overtreatment’ and the impact on the service provision. This is investigated in the Australian setting by Takapautolo et al.,^19^ available in this edition of JMRS. Indeed, the issue of ‘overservicing’ is explicitly noted by The Royal Australian and New Zealand College of Radiologists (RANZCR) and presented as a reason for radiographers to not ‘report’ or indeed provide PIE. Takapautolo et al.^19^ propose that the findings from their large study may support the content focus for education to further improve radiographer PIE accuracy, therefore, potentially reducing the false positive rate. However, based on the findings from this study, it could be argued that in the context of PIE, some degree of ‘overcalling’ in cases of uncertainty may be beneficial as there were an additional 10 patients where pathology was missed by the traditional radiologist report route. The impact of educational intervention for radiographers is further supported in a smaller study by Lewis et al.,^20^ based in New Zealand and published in this edition, where, with regular training and feedback on their image evaluation, radiographer PIE accuracy rose over the course of the study (6 months) from 91.9% to an impressive 96.5%.

## The Use of Artificial Intelligence in Radiology and the Impact on PIE and Reporting

In 2019, the National Health Service (NHS) in the UK introduced their long‐term plan.^21^ This recognised the current plight of the NHS and proposed some strategies to help alleviate the pressure long term and increase the quality of the service. The Plan noted that one key area for development was in the incorporation of technology in patient treatment and care.

The articles referenced in this editorial demonstrate that radiographers are not yet confident on the provision of written PIE, and accuracy rates, whilst impressive, are not perfect. One wonders what the impact of AI will be for these clinicians? With an AI model provided to give a ‘second opinion’, might confidence increase, and accuracy follow? A recent study by the author of this editorial and a team in the UK found that reporting radiographers would be more certain of their diagnosis if they had agreement from an AI tool and that disagreement would drive them to seek a second opinion.^22^ The provision of these technologies in the initial stages of image review are not new, indeed some technology companies have developed apps which allow for the bedside assessment of plain projection chest radiographs for initial interpretation before a definitive report is generated.^23^ With studies indicating that confidence in decision‐making may be a particular issue with radiographers in provision of PIE, and indications that AI assistance may help alleviate this, perhaps the use of technology and radiographer together may shift the paradigm and have impact on how radiographer PIE and by extension, reporting, is viewed by radiologists. However, there should be cognisance of the interaction and development of the relationship between human and machine. Studies suggest that where those who may feel less confident in their decision, such as less experienced clinicians, may relay on the feedback provided by the AI that they are using, even if this contradicts their own judgement.^22^, ^24^ This is known as Automation Bias and may impact users adversely in the initial stages of implementation; however, engagement with adequate education and training may alleviate this somewhat.^22^, ^24^, ^25^, ^26^

## Conclusions

As a profession we need to recognise the unique position clinical radiographers are placed – between technology and patient. Health care systems are struggling around the world and skilled radiographers can be utilised to relieve some of the burden. Whilst the role and development of the radiographer varies internationally, PIE is one area which all radiographers should be in a position to step in; however, this is not being fully implemented, even in the UK. The studies published in this edition of JMRS, in Australia and New Zealand by Brown, Takapautolo and Lewis^13^, ^19^, ^20^ demonstrate that radiographers are competent and that reasons for reticence, such as workload, do not seem to have an impact on performance. However, regular education and feedback may be needed to support this. AI may also have a role in the immediate provision of this feedback but should be approached with caution in an inexperienced group. With time and development of the human‐AI relationship, this may provide a useful support for radiographers working ‘at the front line’, allowing radiographers' confidence in their interpretation, and potentially accuracy, to increase.

## Conflict of Interest

The author declares no conflict of interest.

## Data Availability

Data sharing is not applicable to this article as no new data were created or analyzed in this study.
